# The role of National Immunization Technical Advisory Groups in advising COVID-19 immunization policy during the pandemic: lessons from the Federation of Bosnia and Herzegovina

**DOI:** 10.3389/fpubh.2023.1193281

**Published:** 2023-06-30

**Authors:** Sanjin Musa, Lisa Jacques-Carroll, Mirza Palo

**Affiliations:** ^1^Institute for Public Health of the Federation of Bosnia and Herzegovina, Sarajevo, Bosnia and Herzegovina; ^2^Sarajevo School of Science and Technology, Sarajevo, Bosnia and Herzegovina; ^3^WHO Regional Office for Europe, Copenhagen, Denmark; ^4^WHO Country Office Bosnia and Herzegovina, Sarajevo, Bosnia and Herzegovina

**Keywords:** National Immunization Technical Advisory Group (NITAG), COVID-19 pandemic, COVID-19 vaccination, evidence-based recommendations, immunization, Bosnia and Herzegovina

## Abstract

A National Immunization Technical Advisory Group (NITAG) is a multi-disciplinary body of experts that provides evidence-based recommendations on immunizations to policy-makers to assist them in making immunization policy and program decisions. NITAGs faced challenges in making evidence-based recommendations for COVID-19 vaccines during the COVID-19 pandemic due to the new vaccine products available in a short time period and limited available data on vaccine effectiveness and vaccine safety. The authors reviewed the process used by the NITAG in the Federation of Bosnia and Herzegovina, called the expert body, to develop COVID-19 vaccine recommendations. The article reviews the evidence that was considered by the expert body when developing 23 recommendations on COVID-19 vaccination and describes the challenges and successes faced by the body. The expert body recommendations led to the successful roll-out of COVID-19 vaccines and provided guidance for COVID-19 vaccination during the pandemic. The expert body plans to improve its work and procedures for developing routine immunization recommendations with the support of the WHO Regional Office for Europe.

## 1. Introduction

A National Immunization Technical Advisory Group (NITAG) is a multi-disciplinary body of experts that provides evidence-based recommendations on immunizations to policy-makers and immunization program managers (1). NITAGs help ensure that decisions about immunizations are independent and evidence-based (2) which can increase public confidence in vaccines. The Global Vaccine Action Plan called for all countries to establish or have access to a NITAG by the year 2020 (3). The World Health Organization (WHO) Regional Office for Europe recommends countries in the region strengthen NITAGs and encourage these groups to develop evidence-based policy recommendations for the introduction of vaccines across the life course (4).

As of 1 March 2023, over 758 million laboratory-confirmed cases and 6.8 million deaths from COVID-19 had been reported worldwide (5) and more than 13 billion doses of COVID-19 vaccine had been administered (6). NITAGs played a crucial role in developing COVID-19 vaccine recommendations during the COVID-19 pandemic when there was limited evidence on COVID-19 vaccine safety, vaccine efficacy, and disease epidemiology (7).

The Federation of Bosnia and Herzegovina (FBiH), one of the two entities comprising Bosnia and Herzegovina, has a NITAG (expert body) that has been in existence for almost two decades and played an essential role during the COVID-19 pandemic. FBiH recorded several waves of high SARS-CoV-2 transmission during the pandemic with 264,517 laboratory-confirmed cases and 9,100 deaths (8) and struggled to purchase adequate supplies of COVID-19 vaccines and to reach adequate vaccination coverage. COVID-19 vaccination began in FBIH in early March 2021.

The aim of this article is to describe the role of the FBiH expert body in developing recommendations for COVID-19 immunization policy, the challenges and successes, and future plans.

## 2. Methods

A qualitative retrospective review of the structure and functioning of the expert body and the process used to develop COVID-19 vaccine recommendations was conducted in January 2023. Data were collected through a desk review and interviews with key informants, including two members of the expert body and one staff member from the Ministry of Health (MoH). Key questions asked of expert body members included whether they felt they had enough data to develop COVID-19 vaccine recommendations, if the process for developing recommendations worked well during the pandemic, and what additional support or information would have been helpful when developing COVID-19 vaccine recommendations. A desk review was conducted to gather information which included the expert body rules of procedure, minutes from expert body meetings detailing all decisions on COVID-19 vaccination, and findings from a recent evaluation of the functioning of the expert body by the WHO Regional Office for Europe. The Secretariat also provided an analysis of COVID-19 vaccine uptake in FBiH.

## 3. Results

### 3.1. Establishment of the expert body

In 2005, a NITAG was established in FBiH by the Federal MoH under the Law on Protection from Infectious Diseases (“Official Gazette of FBiH” 29/05) with the title of Expert Advisory Body for Immunization (expert body). The purpose of the expert body is to provide recommendations for immunization policy to the Federal MoH.

### 3.2. Expert body structure and composition

The MoH appoints national experts to serve as members of the expert body. The composition of the expert body was amended by a decree in November 2021, during the COVID-19 pandemic, to expand the number of members from 10 to 12. The expert body currently includes 12 experts with voting rights, including one Chair. The disciplines represented in the expert body include pediatrics, public health, infectious disease, epidemiology, immunology, and pharmacology. The term for members and the Chair is 4 years but re-elections are possible with no limit to the number of re-elections. A member of the Secretariat also serves as a voting member. The expert body invites ex-officio or liaison members to meetings as needed based on topics to be discussed.

### 3.3. Functioning of the expert body

The FBiH expert body has rules of procedure that define the functioning of the group but lacks standard operating procedures. The expert body meets a minimum of twice per year but met more often during the COVID-19 pandemic. The Secretariat organizes the committee meetings, develops agendas, conducts literature reviews, provides data and information, drafts meeting minutes, and drafts and finalizes the meeting reports detailing expert body recommendations. Agendas and information are distributed to members prior to meetings. Expert body decisions are made through voting; a majority of members must be present for decision-making and decisions are made using a simple majority of votes. The capacity of the Secretariat is limited and is provided by a member of the Epidemiology Department of the Institute of Public Health of FBiH. When making routine vaccine recommendations, the expert body relies mainly on WHO Strategic Advisory Committee of Experts on Immunization (SAGE) recommendations.

In 2022, an evaluation of the expert body was conducted with technical assistance from the WHO Regional Office for Europe and Robert Koch Institute (RKI), and an improvement plan was developed. The MoH and the Institute for Public Health of FBiH plan to update the rules of procedures and develop standard operating procedures for the expert body.

### 3.4. Expert body experience developing recommendations on COVID-19 vaccination during the pandemic

In 2020, expert body meetings were initiated by the FBiH Institute of Public Health and MoH during the development of the COVID-19 vaccination plan. The first expert body meeting to discuss COVID-19 vaccination was held on 10 December 2020 and the main topic of discussion was the prioritization of COVID-19 vaccines for the initial roll-out. The expert body met seven times in 2021 and four times in 2022; two of these meetings did not include COVID-19 vaccination topics. In December 2020, the expert group recommended initial priority groups for COVID-19 vaccination but had to meet again in February and April of 2021 to prioritize and re-emphasize the groups eligible for vaccination due to the constrained vaccine supply. Table 1 summarizes the COVID-19 vaccine recommendations that were developed by the expert body from 2020 to 2022; all were accepted by the MoH.

**Table 1:** Recommendations developed by FBiH expert body on COVID-19 immunization policy from 2020 to 2022.

Date	Main issues discussed by expert body	Recommendations
10 Dec 2020	Priority groups for COVID-19 vaccination	Initial priority groups: health workers and health institution staff, persons ≥65 years in social institutions and their caregivers, persons ≥60 years and persons with chronic conditions, and persons in service roles (i.e., police, public transportation, education)
	COVAX and EU4Health procurement mechanisms	Support the use of these mechanisms for procurement of COVID-19 vaccines
	Special regime of cold chain for some COVID-19 vaccines	Increase capacity for ultra-cold chain, coordinate procurement of ultra-cold chain equipment, and designate vaccination sites for COVID-19 vaccines that require ultra-cold storage
5 Feb 2021	Sub-prioritization of recommended groups for COVID-19 vaccination during conditions of limited vaccine supply	Age-based prioritization during conditions of limited vaccine supply; after vaccination of health workers and health institution staff, vaccination should be made available to people ≥75 years of age. Persons with confirmed SARS-CoV-2 infection can postpone vaccination until six months post-infection
	Pfizer/BioNTech and AstraZeneca COVID-19 vaccines	Use of Pfizer/BioNTech and AstraZeneca COVID-19 vaccines for those currently eligible for vaccination
9 Apr 2021	Review and update recommendations for priority groups for COVID-19 vaccination during conditions of limited vaccine supply	Additional age-based prioritization for COVID-19 vaccination during conditions of very limited vaccine supply, focusing on reducing serious disease and mortality. Persons ≤75 years of age considered vulnerable due to a clinical condition should be offered vaccination together with the 60-74-year-old group. Immunocompromised persons should continue to follow infection prevention measures
	CoronaVac COVID-19 vaccine	Use of CoronaVac COVID-19 vaccine for persons ≥18 years of age
	Vaxzevria COVID-19 vaccine	Persons who receive Vaxzevria vaccine should be fully informed about the benefits and risks of vaccination with Vaxzevria vaccine prior to vaccination, including the risk of rare adverse events of thrombosis/thrombocytopenia, how to monitor for symptoms, and what actions to take if symptoms occur
7 May 2021	SARS-CoV-2 Vaccine (Vero Cell), Inactivated (Sinopharm)	Use of SARS-CoV-2 Vaccine (Vero Cell), Inactivated (Sinopharm) for persons ≥18 years of age
	COVID-19 vaccination of persons with previous SARS-CoV-2 infection	Vaccination with one dose of COVID-19 vaccine 3–6 months after a person has recovered from COVID-19 infection; an exception is immunosuppressed persons who should be vaccinated with two doses. Persons who receive the first dose of COVID-19 vaccine and develop SARS-CoV-2 infection with a PCR-positive test in the days after vaccination should receive the second dose 3–6 months after infection
5 Aug 2021	Spikevax COVID-19 vaccine	Use of Spikevax COVID-19 vaccine for persons ≥12 years of age
	Interval between COVID-19 vaccine doses during spread of the Delta strain of SARS-CoV-2	Second dose of COVID-19 vaccine should be administered as early as possible within the recommended interval due to the spread of the Delta variant of SARS-CoV-2 in the FBiH
	Additional recommendations for COVID-19 vaccination with mRNA or Vaxevria vaccines for persons with previous SARS-CoV-2 infection	One dose of mRNA or vector-based COVID-19 vaccine should be administered 3–6 months after a person has recovered from COVID-19 infection (this does not apply to other types of vaccines)
7 Oct 2021	Johnson & Johnson/Janssen COVID-19 vaccine	Use of Johnson & Johnson/Janssen COVID-19 vaccine for persons ≥18 years of age. Persons should be fully informed about the benefits and risks of vaccination with Johnson & Johnson/Janssen COVID-19 vaccine prior to vaccination
	Third dose of COVID-19 vaccine for immunocompromised persons	Third dose of COVID-19 vaccine should be administered to persons ≥12 years of age with severe immunosuppression, with a minimum spacing of 2 months after the second dose; mRNA vaccine is preferred for the third dose
	COVID-19 vaccination of pregnant and breastfeeding women	Pregnant women who choose COVID-19 vaccination should be provided information about the risks of COVID-19 during pregnancy, the benefits of vaccination, and existing safety data. mRNA vaccines are preferred for pregnant women. Breastfeeding women can receive COVID-19 vaccines
	COVID-19 vaccination of children 12–17 years of age	COVID-19 vaccination for children ≥12 years of age with comorbidities using mRNA vaccines
	Co-administration of COVID-19 and influenza vaccines	COVID-19 vaccine and inactivated influenza vaccine can be administered at the same time
5 Nov 2021	Expanded use of a third dose of COVID-19 vaccine	Third dose of COVID-19 vaccine for people who are at highest risk of serious disease and other groups according to previously defined priorities. The third dose should be offered no earlier than 6 months after completion of the primary series. A COVID-19 vaccine that is different from the vaccine used for the primary series may be used. Persons ≥60 years of age who received inactivated vaccines against COVID-19 should be vaccinated with a third dose of COVID-19 vaccine 3–6 months after the second dose
	Expanded COVID-19 vaccination of children aged 12–17 years	All children aged 12–17 years should be offered COVID-19 vaccination. Prior to vaccination, parents/guardians should be fully informed about the benefits and risks of COVID-19 vaccination and consent obtained
23 Dec 2021	First COVID-19 booster dose	Persons ≥18 years of age can receive a booster dose of COVID-19 vaccine 3 months after the completion of their primary vaccination series.
16 May 2022	Second COVID-19 booster dose	Second booster dose at least 4 months after the last dose for: persons ≥75 years of age (especially those residing in long-term care institutions), all persons aged 60–75 years, and persons ≥12 years of age who are immunosuppressed. The use of mRNA vaccines is recommended
13 Sept 2022	Bivalent COVID-19 booster dose	Use of bivalent COVID-19 vaccine adapted to the Omicron variant for first or second booster dose for all vulnerable groups at risk for severe disease and health care workers. The booster dose is recommended a minimum of 3 months after the previous dose

The main evidence considered by the expert committee when developing the initial prioritization groups for COVID-19 vaccination was the WHO SAGE roadmap for prioritizing the use of COVID-19 vaccines in the context of limited supply (9). However, the FBiH expert body felt that the WHO SAGE recommendations developed in 2021 on COVID-19 vaccination were not timely (10). When developing COVID-19 vaccination policy recommendations (e.g., vaccination after COVID-19 infection, spacing between doses, vaccination of children, coadministration of COVID-19 and influenza vaccines, and the administration of booster doses), the expert committee relied mainly on the recommendations of other NITAGs, like the United Kingdom’s Joint Committee on Vaccination and Immunization (JCVI) (11), the United States’ Advisory Committee on Immunization Practices (ACIP) (12), and articles published in peer-reviewed journals for evidence. The WHO prequalification of COVID-19 vaccines (13) and the supporting documents, detailing the data on which WHO based their prequalification of vaccines were important evidence the expert body considered when developing recommendations for different COVID-19 vaccines. The evaluation of COVID-19 vaccines by the European Medicines Agency (EMA) (14) was also considered by the expert body. During the pandemic, the expert body developed recommendations for six different COVID-19 vaccines as well as the updated bivalent booster vaccines.

### 3.5. COVID-19 vaccination uptake in FBiH

**Figure 1:**
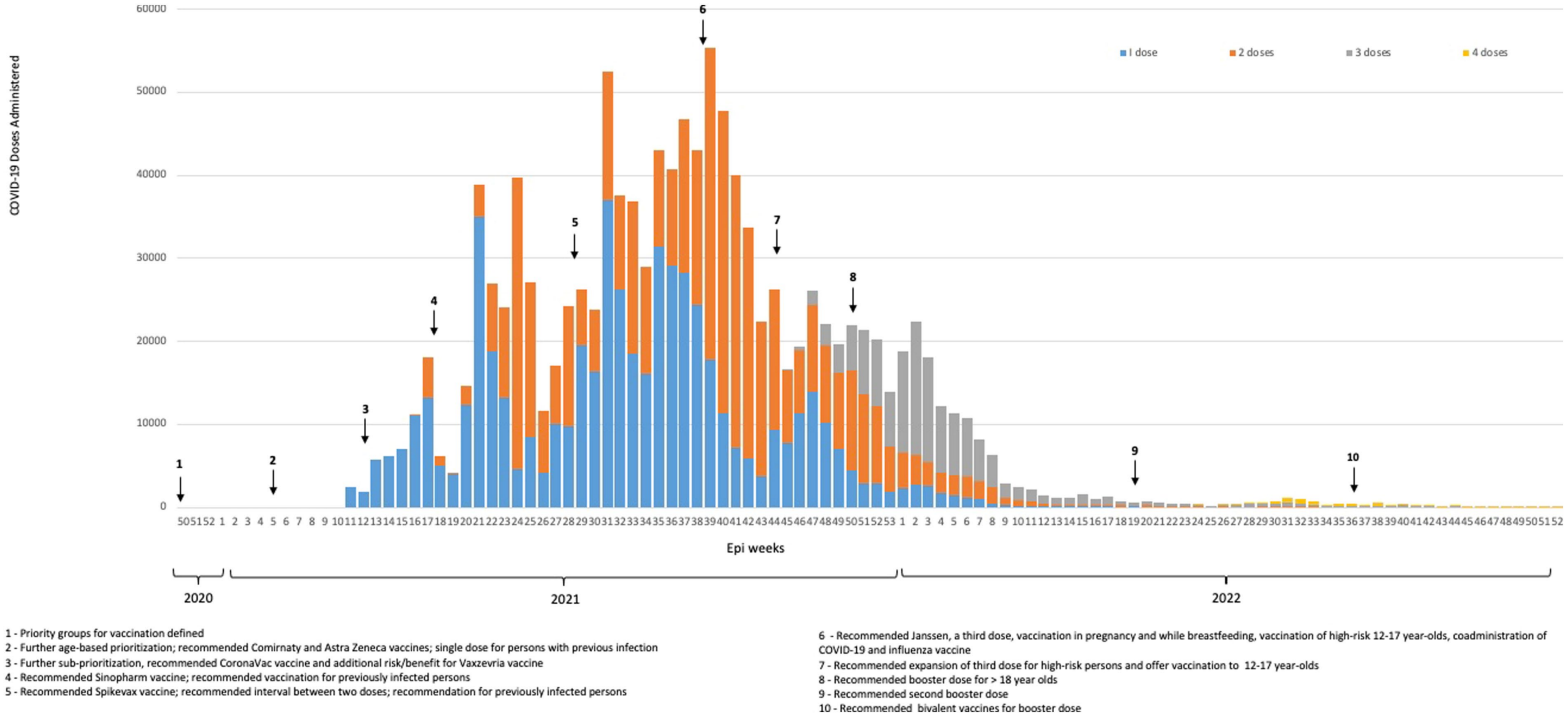
COVID-19 vaccine administration in FBiH and key expert body recommendations, 2020–2022.

FBiH procured COVID-19 vaccines that were developed using a variety of different platforms; initially, vector-based and inactivated vaccines were procured. FBiH first purchased COVID-19 vaccines from COVAX, a global procurement mechanism put in place to supply COVID-19 vaccines to countries, which had limited vaccine supplies and significant delays in COVID-19 vaccine shipments during the pandemic. As some analysis suggests, the global distribution of COVID-19 vaccines has been inequitable (15, 16). FBiH ordered 800,000 doses of COVID-19 vaccines through COVAX but has received only 212,660 doses as of 1 June 2023. The most significant COVID-19 vaccine supply constraints were during the first half of 2021 but vaccine donations were received from Serbia, Türkiye, China, Croatia, and Malaysia during this critical time. More COVID-19 vaccines became available in the second half of 2021 through donations (mostly from European Union (EU) countries), direct procurement from a manufacturer, and procurement through the European Commission’s EU4Health program. The most administered vaccines in FBiH were Comirnaty, Vaxzevria, and Sinopharm (8). Figure 1 shows COVID-19 vaccine administration in FBiH from 2020 through 2022 and indicates when key expert body recommendations were made.

COVID-19 vaccine uptake increased slowly in FBiH during the first 3 months of the roll-out mainly due to constrained vaccine supply. Vaccine uptake increased in mid-2021, once vaccine supplies increased and additional groups became eligible for COVID-19 vaccination and peaked in early September 2021. By the end of 2022, 29.4% of residents aged 18 years or older had received two doses of COVID-19 vaccine, first booster dose coverage was 7.1%, and second booster dose coverage was 0.3% (8). However, official data likely underestimate COVID-19 vaccination coverage due to the vaccination of FBiH residents in neighboring countries and challenges calculating accurate denominators due to emigration from BiH and the lack of recent census data.

A study conducted in 2020 in FBiH found that trust in health professionals and institutions, the COVID-19 vaccine being recommended by the MoH, a greater perception of COVID-19 risk, and being older were among the predictors of a positive response to COVID-19 vaccination. Whereas, being female, the country of COVID-19 vaccine production, and feeling that the pandemic was overhyped by the media were predictors of a negative response to COVID-19 vaccination (17).

## 4. Discussion

### 4.1. Successes and challenges in developing COVID-19 vaccine recommendations

The FBiH expert body experienced challenges in developing COVID-19 vaccine recommendations due to the new COVID-19 vaccine products and limited data on vaccine effectiveness and safety. The expert group had to meet twice in early 2021 to further prioritize the groups eligible for vaccination due to the constrained COVID-19 vaccine supply in FBiH.

WHO SAGE recommendations are essential resources that provide global immunization recommendations. A 2021 global survey of NITAGs found the two most common issues considered during COVID-19 vaccine recommendation-making were the prioritization of the population and vaccine safety concerns (18). The survey also found that policy guidance from SAGE was lacking for certain topics related to COVID-19 vaccination at the time of the survey, including guidance on booster doses and the use of heterologous schedules. Interactions with other NITAGs and improved access to WHO global-level recommendations and evidence were found to be significant aids to NITAGs in developing COVID-19 vaccination recommendations during the pandemic.

The expert body relied mainly on the recommendations of other NITAGs and their supporting materials, local disease surveillance data, and articles published in peer-review journals as sources of evidence when developing COVID-19 vaccine recommendations. At times, the expert body, and NITAGs globally, had to develop recommendations using limited information and evidence on the new COVID-19 vaccines; this experience was shared by NITAGs in the European region during the pandemic (7).

The Global NITAG Network (GNN) (19) is a global network of NITAGs that promotes sharing of resources and experiences through its NITAG Resource Center (NRC) (20) platform. These global resources were especially important during the COVID-19 pandemic when the timely exchange of information and resources was imperative. However, there is also a need to strengthen and expand the GNN and NRC, and countries with stronger NITAGs should be encouraged to share materials and supporting documents used to develop vaccine recommendations in a timelier manner for NITAGs with limited capacity to use and adapt.

Despite the many challenges during the COVID-19 pandemic, the FBiH expert body developed 23 recommendations on COVID-19 vaccination by the end of 2022, all of which were accepted by the MoH. The expert body achieved its mission to support health authorities by providing evidence-based recommendations on immunizations and balancing ethical considerations during a public health crisis and during a time of constrained vaccine supply.

### 4.2. Future plans for FBiH expert body

The WHO Regional Office for Europe has developed an adapted Evidence to Recommendation Process (21) for newly established NITAGs to use when developing recommendations on vaccination policy. However, this adapted process is still time and resource-intensive and may be challenging for NITAGs with limited capacity to implement without significant technical support and/or additional human resources. The Institute for Public Health of FBiH will work with the WHO Regional Office and RKI to establish a process for developing recommendations that will fit the country’s context. The MoH and the Institute for Public Health of FBiH will advocate for additional financial and human resources to support the work of the Secretariat and expert body and will work continuously to increase the visibility and public awareness of the expert body and its recommendations.

## 5. Conclusion

This review highlights the key role the FBiH expert body played, especially during the COVID-19 public health crisis, in developing immunization recommendations. Developing a strong NITAG should be a priority for countries, and one of the most important requirements is an investment in human resources and strengthening the capacity of the Secretariat within public health institutes. This review reinforces the need for improving information sharing and communication between NITAGs through the established networks and with SAGE and other regional advisory groups of experts. Implementing the improvement plan for the FBiH expert body will further build its capacity, improve its functioning, and help the expert body respond to future health crises.

## Data availability statement

The original contributions presented in the study are included in the article/supplementary material, further inquiries can be directed to the corresponding author.

## Author contributions

SM and LJ-C designed the overall concept for the article. LJ-C, SM, and MP wrote the article and SM provided the data and graphics. All authors contributed to the article and approved the submitted version.

## Conflict of interest

The authors declare that the research was conducted in the absence of any commercial or financial relationships that could be construed as a potential conflict of interest.

## Publisher’s note

All claims expressed in this article are solely those of the authors and do not necessarily represent those of their affiliated organizations, or those of the publisher, the editors and the reviewers. Any product that may be evaluated in this article, or claim that may be made by its manufacturer, is not guaranteed or endorsed by the publisher.
